# Elevated Urinary Levels of Fungal and Environmental Toxins in Patients with Pancreatic Ductal Adenocarcinoma

**DOI:** 10.1007/s12029-024-01125-4

**Published:** 2024-10-17

**Authors:** Vanessa I. Rodriguez, Jamila Mammadova, Jennifer B. Permuth, Anjuli Luthra, Luis Pena, Mark Friedman, Aamir Dam, Saraswathi Cappelle, Mokenge P. Malafa, Candice Hallmon, Cassandra Miranda, Shaffer R.S. Mok

**Affiliations:** 1 Department of Internal Medicine, Vanderbilt University Medical Center, Nashville, TN USA; 2 Department of Gastrointestinal Oncology, Moffitt Cancer Center, Tampa, FL USA; 3 Department of Cancer Epidemiology, Moffitt Cancer Center, Tampa, FL USA

**Keywords:** Pancreatic ductal adenocarcinoma, Pancreatic cancer, Fungal toxins, Environmental toxins, Cancer screening, Cancer, Pancreas

## Abstract

**Background:**

Risk factors for pancreatic ductal adenocarcinoma (PDAC) include tobacco/alcohol abuse, genetic predisposition, insulin resistance, and pancreatic cysts. Despite these well-established risk factors and the screening of high-risk individuals, some people still develop PDAC. This study aims to explore a potential risk factor for PDAC by investigating the association between fungal toxins (FT) and environmental toxins (ET) and the disease. We predicted that individuals with PDAC would have higher levels of these toxins compared to healthy controls. The rationale behind this hypothesis is that exposure to FT and ET might contribute to the development of PDAC by elevating cancer risk.

**Methods:**

A pilot retrospective cohort study was conducted at Moffitt Cancer Center from 2022 to 2023. This study compared FT and ET levels, demographic data, and PDAC features between subjects with PDAC and healthy controls.

**Results:**

Forty subjects were enrolled in the study, comprising 20 with pancreatic ductal adenocarcinoma (PDAC) and 20 healthy controls. Baseline demographics were similar between the two groups. Among the PDAC subjects, the most common tumor location was the head of the pancreas (55%); 30% had locally advanced disease, 45% were borderline resectable, and 10% had metastatic disease.

Compared to the controls, subjects with PDAC had significantly higher levels of fungal toxins (FTs) including ochratoxin, gliotoxin, and citrinin (*p* < 0.05). Additionally, PDAC patients had significantly elevated levels of environmental toxins (ETs) such as methyl tert-butyl ether (MTBE), xylene, styrene, acrylonitrile, perchlorate, diphenyl phosphate, bromopropane, organophosphates, acrolein, tiglylglycine, and diethylphosphate (*p* < 0.05).

**Conclusion:**

Our study demonstrates that subjects with PDAC, without other risk factors, have higher FT and ET levels than controls. Further studies are needed to evaluate whether ET and FT exposure can be clinically utilized as a risk factor for PDAC development.

**Supplementary Information:**

The online version contains supplementary material available at 10.1007/s12029-024-01125-4.

## Introduction

Pancreatic ductal adenocarcinoma (PDAC) is the most common type of pancreatic cancer that accounts for more than 90% of all pancreatic cancer cases [1]. It is a highly aggressive malignancy with a historic 5-year survival rate of less than 10% that recently increased to 12%.[2, 3]Early diagnosis of PDAC remains challenging, which contributes to limited treatment options, i.e., inoperability and limited treatment responses.

Screening for PDAC on a population level is hindered by its low incidence, with a lifetime risk of approximately 1.7%, and the absence of simple, non-invasive, and cost-effective screening tools [4]. The American Gastroenterological Association (AGA) recommends targeted screening only for high-risk individuals, particularly those with a family history or predisposing genetic syndromes [5]. AGA guidelines suggest screening high-risk individuals at 50, or 10 years earlier than the initial age of familial onset. Recommended screening age also varies for specific predisposing genetic syndromes and mutations. The preferred screening modality is a combination of magnetic resonance imaging (MRI) and endoscopic ultrasonography (EUS) performed annually with a goal to detect pancreatic neoplasm or precursor neoplasms.

Late detection remains a significant barrier to improving PDAC treatment outcomes, emphasizing the importance of understanding risk factors for effective screening. Recognized risk factors fall into modifiable and non-modifiable categories. Modifiable factors include smoking, obesity, diabetes, chronic pancreatitis, and occupational exposure to specific chemicals used in dry cleaning and metalworking industries [6, 7]. Non-modifiable factors include age, male gender, African American race, family history, inherited genetic syndromes, and chronic pancreatitis due to inherited gene mutation [4, 7, 8]. The impact of certain dietary factors, physical inactivity, alcohol use, and infections (H. pylori, HBV) on PDAC development remains unclear.

Limited studies have explored the associations between environmental toxins (ET) and fungal toxins (FT) exposure and PDAC risk [6, 9–11]. This study aims to investigate whether urine levels of ET and FT can serve as risk indicators for identifying high-risk populations for PDAC, who are otherwise healthy. The hypothesis posits higher levels of specific ET and FT in the PDAC group compared to healthy controls.

## Methods

### Design, Database, and Study Population

A pilot retrospective cohort study was conducted at Moffitt Cancer Center, an academic tertiary cancer center in Tampa, FL, USA, from 2022 to 2023. Subjects were enrolled at baseline, and demographic data, along with information on PDAC including imaging, stage, histology, and endoscopic ultrasound data, were extracted from electronic health records.

All samples were collected at baseline, before any therapies, endoscopies, or anesthesia. Patients fasted from midnight before sample collection, with urine samples obtained upon waking in the morning. The urine specimens were immediately placed on ice and sent to Mosaic Diagnostics in Overland Park, KS, USA, for analysis. The specimens were analyzed using an advanced mass spectrometer for trace levels of mitochondrial, genetic, and toxin compounds.

The study analyzed 11 fungal toxins (FTs)—aflatoxin-M1, ochratoxin A, gliotoxin, sterigmatocystin, mycophenolic acid, roridin E, verrucarin A, enniatin B, zearalenone, chaetoglobosin A, and citrinin (dihydrocitrinone DHC)—and 173 environmental toxins (ETs) using 18 metabolites, including 2-hydroxyisobutyric acid (2HIB), monoethylphthalate (MEP), 2,3,4-methylhippuric acid (2,3,4-MHA), phenylglyoxylic acid (PGO), N-acetyl phenyl cysteine (NAP), N-acetyl(2-cyanoethyl)cysteine (NACE), perchlorate (PERC), diphenyl phosphate (DPP), 2-hydroxyethyl mercapturic acid (HEMA), N-acetyl(propyl)cysteine (NAPR), N-acetyl(2-hydroxypropyl)cysteine (NAHP), N-acetyl-S-(2-carbamoylethyl)cysteine (NAE), N-acetyl(3,4-dihydroxybutyl)cysteine (NADB), dimethylphosphate (DMP), diethylphosphate (DEP), 2,4-dichlorophenoxyacetic acid (2,4-D), 3-hydroxypropylmercapturic acid (3-HPMA), 3-phenoxybenzoic acid (3PBA), and tiglylglycine (TG). A full list of toxins is provided in Appendix A. All FT and ET data were validated by the testing laboratory. PDAC patients provided urine samples within one month of diagnosis. None of the patients exhibited jaundice, infection, or biliary obstruction.

### Inclusion and Exclusion Criteria

Inclusion criteria were adult subjects (age > 18 years) without a history of tobacco or alcohol use, chronic pancreatitis, overweight or obesity, insulin resistance, family history of pancreatic cancer, episodes of acute pancreatitis, or pancreatic cystic lesions. Participants were required to exercise at least 150 min per week, follow a plant-based diet, and maintain a healthy BMI. Exclusion criteria included a history of obesity (BMI ≥ 30), insulin resistance, intraductal papillary mucinous neoplasms (IPMN) and/or mucinous cystic neoplasms (MCN) of the pancreas, chronic pancreatitis, tobacco and alcohol use, and a family history of genetic predisposition to PDAC. Healthy controls were defined as individuals without a pancreatic mass who met these inclusion criteria.

### Statistical Analysis

Statistical analysis was performed using SPSS Statistics (Chicago, IL, USA). Nonparametric tests were employed, with continuous variables compared using the Student’s t-test and Levene’s test for equality of variances. All statistical tests were two-sided, and p-values < 0.05 were considered statistically significant.

## Results

A total of 40 subjects who met the criteria during the study period were included, 20 subjects with PDAC, and 20 healthy controls. The characteristics and presentation of subjects are reported in Table 1. Baseline demographics did not vary significantly between cases and controls. The mean age for PDAC subjects and control group were 70.3 and 67.3 (*p* = 0.31), respectively. 55% of subjects were men within cases (*n* = 11) and 50% of controls (*n* = 10). BMI values did not significantly differ between groups (*p* = 0.10). All subjects included in the study were Caucasian. PDAC features were compared, and the most common location of tumor amongst the study group was the head of the pancreas [*n* = 11 (55%)], followed by the body [*n* = 5 (25%)], stomach of small bowel lumen [*n* = 3 (15%)], tail lumen [*n* = 3 (15%)], and genu lumen [*n* = 1 (5%)]. PDAC subjects had multiple sites of vascular invasion, the most common being invasion of the superior mesenteric vein [*n* = 9 (45%], and only two subjects had metastasis.

**Table 1:** Subject demographics and pancreatic ductal adenocarcinoma characteristics

Parameter	PDAC	No PDAC	*p*-value
Age	70.3 (45–82)	67.3 (52–80)	0.31
BMI	21.36 (19.4–24.3)	20.65 (18.7–24.1)	0.10
**Sex**			
Male	11 (55%)	10 (50%)	0.5
Female	9 (45%)	10 (50%)	
**Race**			
Caucasian	20 (100%)	20 (100%)	1
**Cancer characteristics**			
**Location**			
Head	11 (55%)		
Uncinate	0 (0%)		
Genu	1 (5%)		
Neck	0 (0%)		
Tail	3 (15%)		
Stomach or small bowel	3 (15%)		
Body	5 (25%)		
**Vascular Invasion**			
Celiac	6 (30%)		
Superior mesenteric artery	6 (30%)		
Superior mesenteric vein	9 (45%)		
Hepatic artery	5 (25%)		
Portal vein	8 (40%)		
Portal venous confluence	6 (30%)		
**Metastasis**	2 (10%)		

Age and BMI are reported as averages. All other values are reported as the number of subjects (% of total number of subjects). PDAC, pancreatic ductal adenocarcinoma; BMI, body mass index

Compared to healthy controls, subjects with PDAC had significantly higher urinary concentrations of 3 FTs: ochratoxin, gliotoxin, and citrinin (*p* < 0.05). All other fungal toxins did not present significant differences between the groups (Table 2). The Student’s t-test demonstrated that subjects with PDAC had significantly higher urinary concentration of 11 ETs: methyl tert-butyl ether (MTBE), xylene, styrene, acrylonitrile, perchlorate, diphenyl phosphate, bromopropane, organophosphates, acrolein, tiglylglycine, and diethylphosphate (*p* < 0.05) (Table 3).

**Table 2:** Comparative statistics for urine concentration of fungal toxins in subjects with pancreatic ductal adenocarcinoma and healthy controls

	Levene’s Test for Equality of Variances		Mean Difference	Std. Error Difference
Toxin	F	Significance		
Ochratoxin	20.256 mg/dL	**< 0.001**	10.49667 mg/dL	4.49 mg/dL
Gliotoxin	10.688 mg/dL	**0.002**	174.505 mg/dL	120.1 mg/dL
Citrinin	38.581 mg/dL	**< 0.001**	32.491 mg/dL	23.68 mg/dL

Bolded p-values indicate statistically significant results.

**Table 3:** Comparative statistics for urine concentration of environmental toxins in subjects with pancreatic ductal adenocarcinoma and healthy controls

Toxin	Significance	Mean Difference	Std. Error Difference
	Two-Sided *p*		
MTBE	**< 0.001**	5090.4 ug/g	407.04 ug/g
Xylene	**< 0.001**	487.5 ug/g	119.6 ug/g
Styrene	**0.03**	45.5 ug/g	20.04 ug/g
Acrylonitrile	**< 0.001**	1.4 ug/g	0 ug/g
Perchlorate	**< 0.001**	-4.1 ug/g	0 ug/g
Diphenyl Phosphate	**< 0.001**	-0.5 ug/g	0 ug/g
Bromopropane	**0.001**	17.1 ug/g	4.77 ug/g
Propylene oxide	0.76	0.8 ug/g	2.58 ug/g
Acrylamide	0.71	2.5 ug/g	6.67 ug/g
Organophosphates	**< 0.001**	2.91 ug/g	0.677 ug/g
Acrolein	**< 0.001**	229.2 ug/g	21.60974 ug/g
Tiglylglycine	**< 0.001**	-0.404 ug/g	0.06713 ug/g
Diethylphosphate	**< 0.001**	1.635 ug/g	0.384 ug/g

Bolded p-values indicate statistically significant results. MTBE, methyl tert-butyl ether

## Discussion

The literature has previously outlined the carcinogenic and mutagenic properties of FT and ET, with an emphasis on their potential impact on health; [12, 13] however, the feasibility of utilizing this exposure for PDAC screening has not been explored until now. In our study, urinary concentrations of three FT, ochratoxins, citrinin, and gliotoxins and 11 ETs, were significantly higher in PDAC subjects than controls, indicating a potential association. This study’s findings represent some of the initial evidence suggesting that the concentration of selected FTs and ETs in urine could serve as a potential screening tool for PDAC within the general population.

Dietary intake is the most common source of FT exposure for humans followed by inhalation and dermal contact. Fungal strains that produce FT, such as Penicillium, Aspergillus, and Fusarium species, are commonly found in crops, grains, and stored animal feed. This explains the high chances of human exposure to FT, which results in the systemic circulation of those mycotoxins within the biological systems [14]. Some FT, such as aflatoxins, ochratoxin, fumonisins, zearalenone, and certain Penicillium toxins, have been identified as carcinogenic [12].

Carcinogenic FTs are both genotoxic and mutagenic, posing significant health risks. They can induce genetic mutations and damage, which may contribute to cancer development. The process of cancer promotion by these toxins can be either gradual or rapid, depending on various factors such as exposure levels and individual susceptibility [15, 16]. Moreover, potential synergism between different FTs may play role in carcinogenesis [16]. Ochratoxin A, one of the most lethal FT for humans, is considered to be a nephrotoxin, liver toxin, potent teratogen, carcinogen, and immune suppressant. Its disruptive effects on cellular biophysiology involve multiple mechanisms, such as interfering with the synthesis of the phenylalanine-t-RNA complex, inhibiting mitochondrial ATP production, and increasing the production of free radicals [17–19]. Previous studies revealed potential associations between ochratoxin and the development of renal, gastric, esophageal, breast, and testicular cancers, not PDAC [20–24]. Citrinin, which is commonly found in rice grains, is considered a nephrotoxin and genotoxin, can act alone or in conjunction with ochratoxin, disrupting normal cellular metabolism [25, 26]. Gliotoxin can also cause mitochondrial damage, induce reactive oxygen species, and cause cellular toxicity [27]. While these three FTs have not been directly associated with PDAC in previous studies, our research provides the first evidence suggesting a potentially significant statistical association and clinical utilization. For our sample population, all three FTs had statistically higher concentration in PDAC subjects compared to healthy controls. This is a new finding that could be further evaluated for PDAC screening.

Long-term exposure to numerous ETs has been associated to various cancers like pancreatic, liver, lung, and kidney cancer. Although the exact mechanism of how toxins cause pancreatic cancer is unknown, researchers suggest toxins may reach the pancreas through the bloodstream or via refluxed bile and exert genotoxic effects [28, 29]. Previous studies have shown the relationship between pancreatic cancer and exposure to various ETs, such as pesticides, asbestos, benzene, chlorinated hydrocarbons, chromium, and nickel [6, 10, 30–33]. A meta-analysis of occupational exposures found an increased risk of pancreatic cancer associated with exposure to chlorinated hydrocarbon solvents and nickel, and suggested a heightened risk from other substances, including chromium and organochlorine pesticides [10]. Additionally, a study at the Mayo Clinic involved patients with PDAC and cancer-free individuals completing identical risk factor questionnaires. These questionnaires included yes/no questions about regular exposure to pesticides, asbestos, benzene, chlorinated hydrocarbons, chromium, and nickel. The study found a significant association between self-reported toxin exposure and an increased risk of pancreatic cancer [9]. 

Exposure to MTBE is likely due to groundwater contamination, inhalation or skin exposure to gasoline or its vapors, and exhaust fumes. MTBE has been demonstrated to cause hepatic, kidney, and central nervous system toxicity, peripheral neurotoxicity, and cancer in animals [34]. Xylenes can be found in common products such as paints, pesticides, perfumes, insect repellents, fuel, and exhaust fumes. They are oxidized in the liver and bound to glycine before being eliminated in urine. High exposures to xylene create an increase in oxidative stress, causing symptoms such as nausea, vomiting, dizziness, and death from central-mediated respiratory depression [35]. Styrene is used in the manufacturing of plastics, in building materials and is found in car exhaust fumes. Occupational exposure due to inhalation of large amounts of styrene adversely impacts the central nervous system, causing muscle weakness, fatigue, and nausea [36]. Acrylonitrile is used to produce acrylic fibers, resins, and rubber. Smoking tobacco and cigarettes is another potential exposure, however not present in our sample. Exposure to acrylonitrile can lead to headaches, nausea, and dizziness [37]. Perchlorate can disrupt the thyroid’s ability to produce hormones. This chemical is used in the production of rocket fuel, missiles, fireworks, flares, explosives, fertilizers, and bleach. Many food sources are also contaminated with perchlorate [38]. 

Diphenyl is a triphenyl phosphate (TPHP) metabolite, which is used in plastics, electronic equipment, nail polish, and resins. Studies have also linked TPHP to reproductive and developmental problems [39]. 1-bromopropane is used for metal cleaning, foam gluing, and dry cleaning. It is a neurotoxin and reproductive toxin that can cause sensory and motor deficits. Chronic exposure can lead to decreased cognitive function and impairment of the central nervous system [40]. Acrolein is an environmental pollutant commonly used as an herbicide and in many different chemical industries. It is also present in burning cigarettes, gasoline, and oil. Acrolein metabolites are associated with diabetes and insulin resistance, which theoretically can link to the pancreas [41]. Tiglylglycine serves as an indicator for mitochondrial disorders resulting from mutations of mitochondrial DNA, which can occur secondary to toxic chemical exposures.

Lastly, presence of diethylphosphate indicates exposure to an organophosphate insecticide. In our study, urine concentration of the 11 listed ETs was statistically significantly higher in subjects with PDAC than in healthy controls (Table 3). In addition to FTs, urine concentration of ETs is another potential method that could be considered for risk factors that place individuals at a higher risk of PDAC and potentially warrant screening in the general population.

This study has several limitations that must be acknowledged. Firstly, the small sample size restricts the robustness of the conclusions drawn. Additionally, recall bias regarding patients’ lifetime exposures to known PDAC risk factors—such as tobacco, alcohol, and dietary/exercise habits—could impact the results. Another limitation is the potential high cost of urine screening tests, which may limit their availability at other institutions. Moreover, the optimal timing and frequency for evaluating urinary concentrations of FTs and ETs for screening purposes require further analysis and development. To address these limitations and assess the generalizability of our findings, additional studies with larger populations are needed. Furthermore, it is unethical to expose individuals to such chemicals to track their risk of PDAC over time. Consequently, while our findings suggest an association, they do not establish causality.

However, this study has several strengths. It is the first to assess FT and ET as risk factors for developing PDAC. Its clinical potential includes offering a simple and convenient method for risk stratification in healthy individuals who do not have traditional risk factors for PDAC. The study also highlights its clinical significance by showing that identifying high toxin levels can lead to interventions aimed at reducing or eliminating these toxins. For example, if organophosphates are detected, individuals can stop using herbicides on their lawns and opt for herbicide-free products. Similarly, for fungal toxins like ochratoxin, inspecting homes for water damage and mold—and addressing any issues—can be a proactive measure.

In conclusion, our study revealed that subjects with PDAC had higher urinary levels of FTs and ETs compared to healthy controls. These findings suggest that urinary toxin concentrations could serve as a potential screening tool for PDAC. However, our study identified an association rather than establishing causality. Further research with larger patient cohorts is needed to confirm whether urinary toxin concentrations could indeed serve as an effective screening tool for PDAC.

## Supplementary Information

Below is the link to the electronic supplementary material.

ESM 1 (DOCX 18.8 KB)

## Data Availability

No datasets were generated or analysed during the current study.
