# Supplementary material for: Elevated Urinary Levels of Fungal and Environmental Toxins in Patients with Pancreatic Ductal Adenocarcinoma
Source: J Gastrointest Cancer. 2024 Oct 17;56(1):4. doi: 10.1007/s12029-024-01125-4 (PMC11486816; doi:10.1007/s12029-024-01125-4)
Supplement: Supplementary file 1 — (DOCX 18.8 KB) [file 12029_2024_1125_MOESM1_ESM.docx]

# Appendix A

## List of tested fungal toxins in alphabetic order:

1. Aflatoxin-M1
2. Chaetoglobosin A
3. Citrinin (dihydrocitrinone DHC)
4. Enniatin B
5. Gliotoxin
6. Mycophenolic acid
7. Ochratoxin A,
8. Roridin E
9. Sterigmatocystin
10. Verrucarin A
11. Zearalenone

## List of 18 tested metabolites in alphabetic order:

1. 2-3-4 methylhippuric acid (2,-3-,4-MHA)
2. 2-hydroxyethyl mercapturic (HEMA),
3. 2-hydroxyisobutyric acid (2HIB)
4. 2,4-dichlorophenoxyacetic acid (2-,4-D)
5. 3-hydroxypropylmercapturic acid (3-HPMA)
6. 3-phenoxybenzoic acid (3PBA)
7. Diethylphosphate (DEP)
8. Dimethylphosphate (DMP)
9. Diphenyl phosphate (DPP)
10. Monoethylphthalate (MEP)
11. N-acetyl phenyl cysteine (NAP)
12. N-acetyl-S-(2-carbamoylethyl) cysteine (NAE)
13. N-acetyl(2-cyanoethyl)cysteine (NACE)
14. N-acetyl(3,4-dihydroxybutyl) cysteine (NADB)
15. N-Acetyl(propyl) cysteine (NAPR) (parent: N-acetyl(2-hydroxypropyl)cysteine (NAHP)
16. Perchlorate (PERC)
17. Phenylglyoxylic Acid (PGO)
18. Tglylglycine (TG)

## List of 173 environmental toxins tested through analysis of 18 metabolites in alphabetic order:

1. (E)-Mevinphos
2. (Z)-Mevinphos
3. 1-bromopropane
4. 1,3 butadiene
5. 5-Dichloro-alpha- (chloro-methylene) benzyl diethyl phosphate
6. Acethion
7. Acetoxon
8. Acrolein
9. Acrylamide
10. Acrylonitrile
11. Akton
12. Amidithion
13. Amiton
14. Amiton oxalate
15. Anilofos
16. Athidathion
17. Azamethiphos
18. Azethion
19. Azinphos
20. Azinphos-ethyl
21. Azinphos-methyl
22. Azinphos-methyl oxygen analog
23. Azothoate
24. Benzene
25. Bomyl
26. Bromophos
27. Bromophos-ethyl
28. Butathiofos
29. Carbophenothion
30. Chlorethoxyphos
31. Chlorfenvinphos
32. Chlorphoxim
33. Chlorprazophos
34. Chlorpyrifos
35. Chlorpyrifos oxygen analog
36. Chlorpyrifos-methyl
37. Chlorthion
38. Chlorthiophos
39. Chlorthiophos II
40. Chlorthiophos III
41. cis-Azodrin
42. cis-Methocrotophos
43. Coumaphos
44. Coumithioate
45. Crotoxyphos
46. Cyanophos
47. Cyanthoate
48. Cyhalothrins
49. Cypermethrin
50. Cythioate
51. Deltamethrin
52. Demephion-O
53. Demephion-S
54. Demeton
55. Demeton-O
56. Demeton-O-methyl
57. Demeton-S
58. Demeton-S-methyl
59. Dialifor
60. Diazinon
61. Diazoxon
62. Dichlofenthion
63. Dichlorvos (DDVP)
64. Dicrotophos
65. Diethyl phthalate
66. Diethyldithio phosphate
67. Diethylthio phosphate
68. Dimethoate
69. Dimethoate-ethyl
70. Dimethyl chlorophosphonate (DMCP)
71. Dioxathion
72. Diphenyl Phosphate
73. Disulfoton
74. Disulfoton sulfone
75. Disulfoton sulfoxide
76. Endothion
77. Ethion
78. Ethion O-analog
79. Ethyl tert-butyl ether (ETBE)
80. Ethylbenzene
81. Ethylene oxide
82. Etrimfos
83. Famphur
84. Famphur O-analog
85. Fenitrothion
86. Fenpropathrin
87. Fensulfothion
88. Fenthion
89. Fenthion oxon
90. Formothion
91. Fosmethilan
92. Fospirate
93. Halopropane
94. Heptenophos
95. Iodofenfos
96. Isazophos
97. Isazophos-methyl
98. Isochlorthion
99. Isothioate
100. Isoxathion
101. Lythidathion
102. Malaoxon
103. Malathion
104. Mecarbam
105. Menazon
106. Methacrifos
107. Methidathion OA
108. Methyl paraoxon
109. Methyl phenkapton
110. Methyl tert-butyl ether (MTBE)
111. Methyl trithion
112. Mevinphos
113. Miral
114. Monocrotophos
115. Morphothion
116. Naled
117. Naphthalophos
118. Omethoate
119. OO-diethyl O-naphthaloximido phosphorothioate
120. OO-diethyl phosphoro chloridothionate
121. OO-Diethyl S- (46-dimethyl-2-pyrimidinyl) phosphorodithioate
122. OO-diethyl-O-phenyl phosphoro thioate
123. OOS-Trimethyl phosphorodithiate
124. Organophosphates metabolized into DEP
125. Organophosphates metabolized into DMP
126. Oxydemeton-methyl
127. Paraoxon
128. Parathion
129. Perchlorate
130. Permethrin
131. Phenkapton
132. Phenthoate
133. Phorate
134. Phosalone
135. Phosmet
136. Phosmetoxon
137. Phosnichlor
138. Phosphamidon
139. Phoxim
140. Phoxim-methyl
141. Pirimiphos ethyl
142. Pirimiphos-methyl
143. Primidophos
144. Propoxon
145. Propylene oxide
146. Prothidathion
147. Prothion
148. Prothoate
149. Pyrazophos
150. Pyridiphenthion
151. Quinalphos
152. Quinalphos-methyl
153. Quinothion
154. Ronnel
155. Sophamide
156. Styrene
157. Sulfotep
158. Temephos
159. Temephos sulfoxide
160. Terbufos
161. Terbufos sulfone
162. Terbufos sulfoxide
163. Tetrachlorvinphos
164. Tetraethyl pyrophosphate (TEPP)
165. Thiometon
166. Thionazin
167. Thionazin O-analog
168. Tolclofos-methyl
169. Triazophos
170. Trihalomethrin
171. Vamidothion
172. Vinyl chloride
173. Xylene
